# A Review of The Lesser-Studied Microemulsion-Based Synthesis Methodologies Used for Preparing Nanoparticle Systems of The Noble Metals, Os, Re, Ir and Rh

**DOI:** 10.3390/ma12121896

**Published:** 2019-06-12

**Authors:** Mohammad Soleimani Zohr Shiri, William Henderson, Michael R. Mucalo

**Affiliations:** School of Science, University of Waikato, Hamilton 3240, New Zealand; m.soleimanizh@gmail.com (M.S.Z.S.); hende@waikato.ac.nz (W.H.)

**Keywords:** microemulsions, micelles, synthesis, noble metals, nanoparticles, osmium, rhenium, iridium, rhodium

## Abstract

This review focuses on the recent advances in the lesser-studied microemulsion synthesis methodologies of the following noble metal colloid systems (i.e., Os, Re, Ir, and Rh) using either a normal or reverse micelle templating system. The aim is to demonstrate the utility and potential of using this microemulsion-based approach to synthesize these noble metal nanoparticle systems. Firstly, some fundamentals and important factors of the microemulsion synthesis methodology are introduced. Afterward, a review of the investigations on the microemulsion syntheses of Os, Re, Ir, and Rh nanoparticle (NP) systems (in all forms, viz., metallic, oxide, mixed-metal, and discrete molecular complexes) is presented for work published in the last ten years. The chosen noble metals are traditionally very reactive in nanosized dimensions and have a strong tendency to aggregate when prepared via other methods. Also, the particle size and particle size distribution of these colloids can have a significant impact on their catalytic performance. It is shown that the microemulsion approach has the capability to better stabilize these metal colloids and can control the size of the synthesized NPs. This generally leads to smaller particles and higher catalytic activity when they are tested in applications.

## 1. Introduction

Nanotechnology can be defined as the synthesis and development of materials of nano-sized dimensions (1 to 100 nm). The experiments that Michael Faraday did on metal colloids (especially gold) provided an initial scientific view into this world of nanotechnology. He used elemental phosphorus to reduce an [AuCl_4_]^–^ solution to produce a red solution of gold colloids (3–30 nm sized particles) [1,2,3,4]. Colloids, the original older term connected to this field, can be defined as the dispersion of one liquid or solid phase in another phase that exists without any spontaneous phase separation or sedimentation (particles in the size range of 1 to 1000 nm) [4]. One example of a colloidal system is emulsions, which form when a liquid disperses in another immiscible liquid medium [5]. Emulsions can also be used to form other colloidal or nanoparticle systems as is detailed in this review.

In general, in nanoparticle science, NPs can be classified based on their overall shape, ranging from zero-dimensional (0D) to three-dimensional (3D). This classification measures the dimensions of nano structural materials (NSMs) which are not measured in nanoscales. Quantum dots, nanowires, nanotubes, nanodisks, and nanoflowers are some examples of 0D to 3D NPs, respectively [6]. 

Nanoparticle synthesis methods can be generally classified via two approaches, i.e., top-down and bottom-up. In the top-down method, larger scale structures decompose to nanostructures and vice versa for the bottom-up method which starts with the constituent atoms to reach clusters [6,7,8]. Chemical reduction, as a bottom-up subclass synthesis method, is a classical and established NP preparation method which is applied in the preparation of a variety of metal and metal oxide NPs, e.g., silver, gold, copper, Re_2_O_7_, and Cu_2_O [9,10,11,12,13,14]. In this method, metal salts and other compositions are reduced chemically by a reducing agent to achieve NPs. Controlling the reaction to reach a desired size and shape, usually by adjustment to the reaction conditions and constituents, is an available strategy in this bottom up synthesis method [15].

### 1.1. Noble Metal NPs

Noble metals include gold (Au), silver (Ag), platinum (Pt), palladium (Pd), ruthenium (Ru), rhodium (Rh), rhenium (Re), osmium (Os), and iridium (Ir). In the last two decades, the investigation of novel and promising syntheses of noble metal NPs has been pursued due to strong interest for two major reasons: (1) the growth in use of noble metal NPs in various applications; and (2) different shapes, sizes and compositions of noble metal NPs can introduce new and sometimes tailorable properties [15,16]. The morphology and size dependence of noble metal NP applications have been well documented [17]. The all-important effect of the particle size reduction is to increase the catalytic applications of noble metal NPs by virtue of the large increase in the surface area caused by the exposure of more atoms on the surface (as smaller particle size equals more catalytically active sites). NP/colloid scientists wish to develop noble metal synthesis methods to achieve better control of size and shape and to prepare more monodisperse and stable NPs to enhance their performance in the applications they have been prepared for [18]. In this paper, we review recent developments in the synthesis of some noble metal NPs (Re, Ir, Rh, and Os) via microemulsion-based methodologies as these media have the capability to stabilize, allow control of particle sizes, and in some cases, alter the morphology of the prepared NPs.

### 1.2. Fundamentals of The Microemulsion Technique

Microemulsions can be described as a thermodynamically stable system of two immiscible liquids. The dispersed phase, which is present at a lower volume, forms the droplets [19] in a microemulsion. These systems depend on the nature of the dispersed liquid so that dispersion liquids may be classified as either oil in water (O/W), where oil droplets exist dispersed in bulk water, or conversely, water in oil (W/O), where water droplets are dispersed in oil. There are also oil in oil (O/O) systems, where two (polar and non-polar) oils mix together [20]. Microemulsions also are isotropic and transparent (or translucent) systems, which consist of at least three components: a polar phase, a non-polar phase, and a *surfactant* [21,22,23].

**Figure 1:**
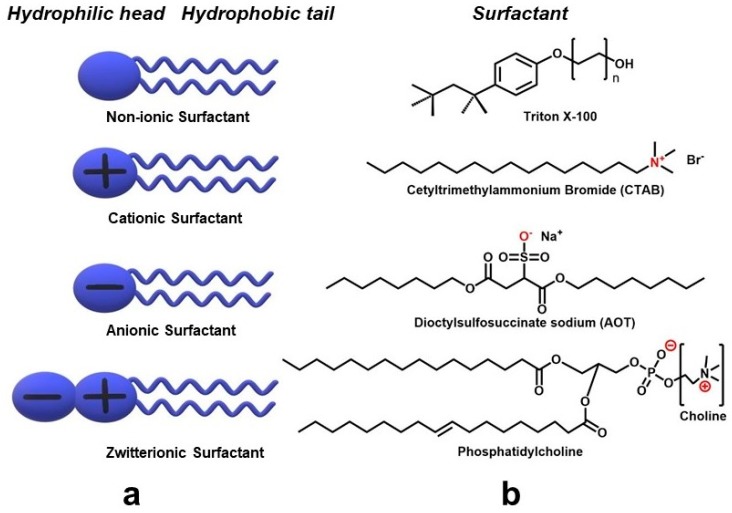
(**a**) A schematic of different type of surfactants and (**b**) Chemical structures of some common surfactants that have been used in a microemulsion synthesis of nanoparticles (NPs).

Surfactants in such microemulsion systems act as the emulsifier. These amphiphilic molecules are classified (based on the charge of their head groups) as non-ionic, anionic, cationic, and zwitterionic (i.e., carrying both positive and negative groups) surfactants (Figure 1) [24]. Cetyl trimethyl ammonium bromide (CTAB), a cationic surfactant [25,26]; dioctylsulfosuccinate sodium (AOT), an anionic surfactant [27]; phosphatidylcholine (PC) [28], a zwitterionic surfactant; and Triton X-100, a non-ionic surfactant (Figure 1) [29,30] are some common surfactants that have been used in the past to synthesize NPs using the microemulsion method.

**Figure 2:**
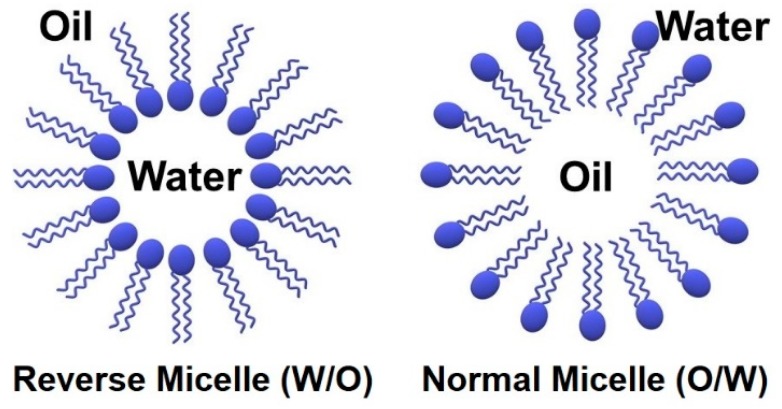
Schematic structure of a reverse and normal micelle.

These surfactant molecules adsorb at interfaces (the boundary between two immiscible phases) and are used to stabilize microemulsions by reducing the interfacial tension and free energy [22,24]. Another characteristic of surfactants is their tendency to aggregate and form micelles after a particular concentration, the critical micelle concentration (CMC) [22,24,31]. In an O/W microemulsion, the hydrophilic head groups of a surfactant molecule are oriented out from the micelle (in order for it to be in contact with water with which it has molecular affinity) and the hydrophobic tails associate within the core (within the entrapped oil droplets), so ‘*normal micelles*’ form and vice versa for a W/O microemulsion, where ‘reverse micelles’ are generated (Figure 2) [21,22]. The core of reverse microemulsions (reverse micelles) can play a significant and active role for NPs synthesis as ‘*nano reactors*’ [21,22]. Water in the core of reverse micelles is called a water pool. Their size is in the nano scale, and this can be controlled by changing some factors such as the water to surfactant ratio (*W*) [21,32]. Generally, decreasing the value of *W* leads to smaller water nano droplets and consequently smaller NPs [21,33].

### 1.3. Preparation of Nanoparticles Using Microemulsion Techniques

**Figure 3:**
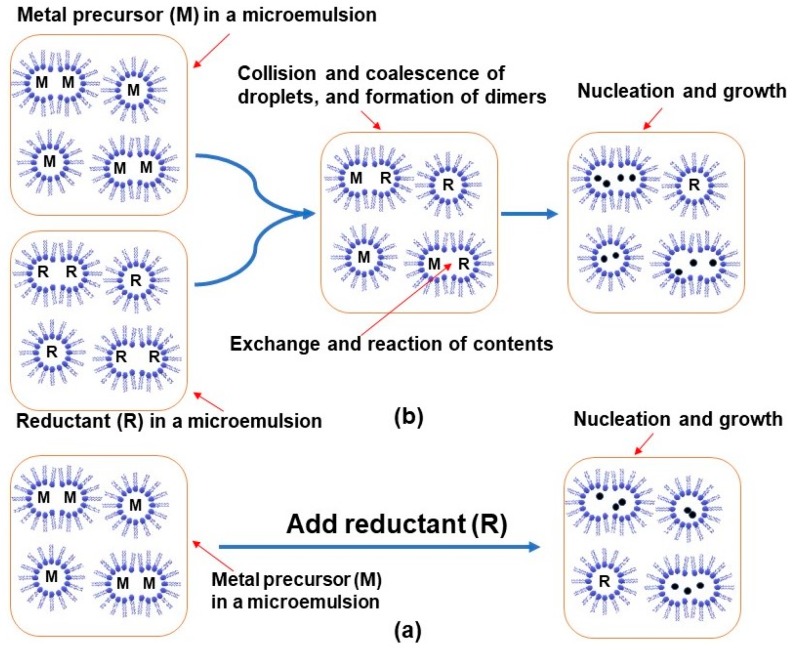
Colloidal synthesis by a microemulsion technique: (**a**) adding directly the reducing agent to the microemulsion containing the dissolved metal precursor; (**b**) mixing two microemulsions containing metal precursor and reductant.

Microemulsions attracted a significant amount of attention as a convenient medium for NPs generation when they were first introduced by Boutonnet et al. in the early 1980s. Boutonnet synthesized some colloidal noble metals in nano water pools by a chemical reduction method [34,35,36]. Microemulsions have some particularly useful properties such as high stability, extremely small droplet size (internal structure), and a specific structure (e.g., spherical) [21,22,33]. In addition, the size and shape of droplets can be controlled by varying the constituents [21,37]. Through these, microemulsions have become a promising medium for the size-controlled synthesis of colloidal particles [21,33]. A reverse micelle (W/O microemulsion) is of considerable interest for metal colloids preparation and is mostly carried out using chemical reduction methods. This is due to the capability of water pools (the interior structure of the micelles) for solubilizing the metal precursors and using them in the reduction process to achieve the synthesis of metal NPs. The size of synthesized NPs can be controlled by changing the size of water pools as these can consequently restrict further growth of the NPs. Also, the composition of certain entities of NPs is able to be altered by changing the metal precursors [21,22,33]. To achieve NPs using the microemulsion technique and reduction methods, two major ways have been used: either (1) mixing the metal precursor in a microemulsion with the reducing agent (Figure 3a); or (2) mixing the metal precursor in a microemulsion with the reducing agent encapsulated in another microemulsion (Figure 3b) [22,33]. With reference to Figure 3a,b, depending on the “flexibility of the surfactant film”, only a limited number of droplet collisions will have enough energy to allow the formation of dimers to exchange the reactants [21,38,39]. If the two microemulsion units (monomers) about to coalesce contain merely different concentrations of the same reactant (e.g., the metal NP precursor molecule), the content of the resulting dimer will only exchange via the concentration gradient. But when the dimer contains the metal precursor and the reducing agent, then, they react and generate the nuclei of NPs in the droplets themselves (Figure 3) [21] to yield the reverse microemulsion-encapsulated NPs.

NP generation in an O/W (normal) microemulsion is uncommon but there still have been some nanostructures synthesized by this technique. In an O/W microemulsion, normal micelles form which generate (in contrast to the water pools within reverse microemulsions) confined oil droplets. Generally, the two ways of introducing the metal precursors for generating the NPs in an O/W microemulsion synthesis system are, (1) the use of an ionic salt precursor dissolved in the aqueous dispersion medium; and (2) the use of an organometallic precursor dissolved in the nano oil droplet pools [40,41].

### 1.4. Factors Affecting NPs Synthesis in a W/O Microemulsion

Several factors affect the NPs size in such a microemulsion synthesis method. These include:

(a) Water and surfactant effects

The amount of water present as the dispersed phase in a W/O microemulsion method has a significant effect on the size of the synthesized NPs. This parameter is usually described by the term *W* which refers to the water-to-surfactant concentration ratio [21,22,42]. Many papers evaluate the effect of *W* on NPs size and report that an increase in *W* leads to an increase in NPs size (mostly when *W* is <10–15) [32,33,43,44,45,46]. This is because at lower water concentration values (*W* < 10–15) surfactants make more tightly packed water pools (smaller micelles), which lead to smaller and more stabilized NPs. Consequently, in the W/O microemulsion-based syntheses of NPs, the strong influence of water concentration (*W*) should be the primary consideration for the achievement of smaller NPs [21,22,33]. 

Several studies have evaluated the effect of surfactant on the NP size and structure when they have been generated in a W/O microemulsion synthesis method as well. Most of them revealed that an increase in the amount of surfactant (at a constant concentration of water and oil) leads to smaller particles by decreasing the *W* value and increasing the number of droplets [22,33]. In addition, by increasing the surfactant concentration, the number of surfactant-surrounded particles will increase. This results in more control of the nucleation process rate (which occurs inside the droplets) and prevents particles from growing too quickly. Eventually, the surfactants direct particles to become smaller and more homogenous in their particle size distributions [22,33]. Therefore, the final size of NPs depends on the size of droplets and also on the number of surfactant-surrounded particles (which affect the size of nuclei, so controlling the agglomeration process and also stabilizing the synthesized NPs) [22,23,33].

Sometimes, a secondary surface-active agent, which is referred to as a co-surfactant, is required to prepare microemulsion systems. Co-surfactants can be defined as weak surfactants that usually have a small polar head group (such as a hydroxyl group) and consequently, do not possess adequate ability to stabilize the microemulsions *per se*. They are mostly medium chain alcohols or amines and, in a mixture with a surfactant, are often used to decrease the interfacial tension and to help microemulsions form and to stabilize [21,22]. He et al. in 2003, evaluated the co-surfactant effect on the size of cerium oxalate NPs as a consequence of increasing stability. They used different alcohols as co-surfactants and reported that through increasing the chain length of alcohols, free energy decreased, and more stable and smaller particles resulted (for instance, particle size decreased from 63.4 nm when butyl alcohol was used to 47.1 nm when heptyl alcohol was used) [47].

(b) Effect of the nature and concentration of reagents used

The concentration of reagents also plays a significant role in the size of NPs synthesized by microemulsion techniques. Most studies reported that the particle size increased in direct proportion to the concentration of reactants [16,21,22,48,49], while some studies showed an opposite effect [33,50]. In 2003, the size of silver NPs was considered by Maillard et al. to be a function of the concentration of reducing agent (hydrazine to AOT ratio). They achieved an increase in particle size by increasing hydrazine concentration [49]. However, in 2000, Chen et al. synthesized Ni NPs in W/O microemulsions and achieved smaller particles by increasing the hydrazine concentration (the size of particles remained unchanged after the hydrazine to nickel chloride ratio was altered until equal to 10) [50]. Lisiecki et al., in 2003, also investigated the effect of reductant amount on the size distribution of cobalt nanocrystals and observed a narrower particle size distribution by increasing the amount of reductant (sodium borohydride) [48]. Also, the nature of reductants can affect the NPs size. Solanki et al. evaluated the influence of different reducing agents on silver NPs using hydrazine hydrate and sodium borohydride as a comparison. They kept the *W* constant at the optimal amount (*W* = 3) and compared the NPs obtained by using hydrazine hydrate (as a reducing agent) as opposed to sodium borohydride. The results illustrated smaller particles when the reduction was done using hydrazine hydrate which is regarded as a weaker reductant than NaBH_4_, and hence reduces the silver ions at a slower rate [51].

## 2. Recent Investigations on The Microemulsion-Based Synthesis of Re, Ir, Os, and Rh NPs

In past works, microemulsion-based synthesis of noble metal NPs has mostly concentrated on noble metals such as Au, Ag, Pt, and Pd. This has probably been due to the variety of uses such as electrocatalytic applications [30,52], methanol production [53], combustion and environmental applications [54,55,56,57], etc. that these microemulsion-prepared NPs have been put to.

In this review, discussion is provided on recent developments in the synthesis of noble metal NPs consisting of the more uncommon Re, Ir, Os, and Rh systems in all forms including metallic, mixed metallic, oxide, and complex molecular forms, that have generally attracted less attention research-wise when prepared by the microemulsion technique. These NPs have different practical uses from biological benefits (e.g., in imaging and cancer therapy) [58,59,60,61], to mainly catalytic and electrocatalytic applications (e.g., hydrogenation of arenes, oxygen reduction in fuel cells, and cyclic acetals synthesis as fuel additives) [62,63,64,65,66]. Interestingly, to our knowledge, there are no previously reported studies for the synthesis of metallic Os NPs by microemulsion methods at the time of writing. There are related studies, however, which use micellar systems. It was only recently, in 2014, that Barry et al. fabricated an Os complex NPs by encapsulating a hydrophobic osmium organometallic complex, Os(p-cymene)(1,2-dicarba-closo-dodecarborane-1,2-dithiolate), into a water-soluble pluronic triblock copolymer P123 micelle, and evaluated their potential anticancer activities. Pitto-Barry et al. followed the investigations of Barry et al. and synthesized Os nanocrystals by irradiating encapsulated Os complexes with high-energy electron beams. Transmission Electron Microscopy (TEM) studies revealed the existence of Os metallic NPs but XPS studies detected no metallic (zero valent) Os (only forms in the Os(II) and Os(IV) oxidation state) which concluded that the Os NPs were oxidized rapidly by exposure to air [67,68,69,70].

There was also a report in 2003 by Ley et al. which detailed the use of an O/W microemulsion for preparing microencapsulated osmium tetroxide that was employed for the dehydrogenation of olefins. However, the particles were not reported to be in true nano-sized dimensions (i.e., they ranged from 20–250 µm) [71]. The other metals to be covered in this review (i.e., Re, Ir, and Rh) have been reported as being prepared via microemulsion-mediated syntheses in the last 10 years, though the numbers of reports are still small by comparison to other more commonly studied noble metals such as Pt, Pd, Au, and Ru. Most recently, microemulsion synthesis research has been conducted for Rh NPs as these systems have many potentially interesting applications such as catalysis of NO reduction (i.e., NO-remediation from car exhausts), increasing the thermal stability of nanocomposites, and other applications that will be discussed below.

### 2.1. Microemulsion-Based Methodologies for Generating Re NPs

Gyger et al., in 2013, developed an interesting and uncommonly used reverse microemulsion system for synthesizing some NPs including Re NPs. They tried to make a water-free medium for synthesizing NPs in which they used ammonia instead of water as the polar phase for making microemulsion systems, and for the first time applied an ammonia in oil microemulsion (“a/o” microemulsion) for preparing NPs. A mix of a double-tailed surfactants, dimethyl dioctyl ammonium bromide or iodide (DDAB or DDAI), with hexyl or heptyl amine as co-surfactant in an emulsion with *W*_NH_3__ (ammonia to surfactant concentration ratio) up to 22 was used for making this a/o microemulsion. The mixture of surfactant/co-surfactant in n-heptane (as the oil phase) and ammonia was turbid and needed to be cooled to −40 °C to clarify the liquid (n-heptane freezing point is −91 °C and liquid ammonia boiling point is −33 °C). Re NPs formed in this a/o microemulsion (by using sodium borohydride as the reducing agent) and were found to have a mean particle size of 2.2 nm as characterized by TEM and High–Resolution Transmission Electron Microscopy (HRTEM). This a/o microemulsion technique was judged useful for the synthesis of metallic NPs that are customarily very sensitive to oxidation and hydrolysis (such as Re metal) [72]. 

In 2013, Revina et al. synthesized Re NPs in a reverse micelle solution using ammonium perrhenate (NH_4_ReO_4_), isooctane, AOT, and an inert gas (Ar) via two methods: 1) γ-irradiation to reduce the “Re^7+^” ions (which one would assume from the paper to be from NH_4_(ReO_4_)); and 2) chemical reduction of NH_4_(ReO_4_) by dihydroquercetin and quercetin (as two polyphenol compounds). UV-Vis and AFM studies confirmed the presence of the Re NPs [73]. Then, in 2018, another paper by the same authors reported a method using similar procedures but with the addition of an extra agent, isopropyl alcohol, in the radiation-chemical method (to generate hydroxyl isopropyl radical as another reducing agent). As TEM characterizations revealed in these studies, adjusting the preparation conditions such as *W* and the reducing method, led to the size of the Re NPs produced varying over the range of 1–18 nm. The authors also studied the effect of irradiation doses and found that decreasing the dose of irradiation in the radiation chemical synthesis method generated smaller particles. Also, as expected, increasing the *W* values led to larger particles [74]. 

Bedia et al., in 2015, synthesized ReNPs with a traditional chemical reduction method combined with a microemulsion technique. They used two rhenium precursors, K_2_ReCl_6_ and NH_4_ReO_4_, with poly vinyl pyrrolidone (PVP), a protecting agent, and NaBH_4_ (the reductant) with the synthesis done under air or an inert atmosphere (with bubbling to remove oxygen from water to prevent rhenium oxidation), in order to prepare ReNPs (0.7–2.8 nm). The microemulsion preparation method used water/AOT/n-heptane, to form smaller and narrower size distribution NPs (0.7–1.4 nm), as characterized by TEM. The effect of *W* was evaluated as well. In contradistinction to most results obtained from microemulsion synthesis research, they obtained a smaller mean size and narrower size distribution of NPs by using a microemulsion system with a higher *W* value (i.e., when *W* varied between 1–8) [32]. They did not apparently analyze the oxidation state of the prepared Re NPs as controlling the oxidation state of these NPs for the XPS sample preparation is challenging as demonstrated in other studies by Mucalo et al. Re NPs highly tend to oxidize slowly and spontaneously to higher oxidation states such as perrhenate ion which contains Re in its +7 oxidation state [14,32,75]. 

Asrami et al. reported a bimetallic form of Pt-Re NPs in 2016 that was supported on γ-alumina by means of a microemulsion technique combined with an impregnation method (impregnation methods usually involve filling the pores of a support by a metal precursor solution and then heating with reduction reagents such as hydrogen gas that can access metal particles lodged in the pores of the alumina support [76]). For the microemulsion-based method, Triton X-100 was used as the non-ionic surfactant, together with n-butanol as the co-surfactant, n-hexane as the dispersion phase, and aqueous solutions of H_2_PtCl_6_ and HReO_4_ as the metal precursors. NaBH_4_ was used as the reducing agent for synthesizing the Pt-Re NPs. They compared the catalytic activity of the bimetallic catalysts prepared by the impregnation and the microemulsion method in naphtha reforming. TEM studies demonstrated that the microemulsion method generated smaller particles (smaller particles formed in preparations carried out with smaller *W* values, from 2.05 nm for *W* = 1 to 1.8 nm for *W* = 0.3) and hence the colloid preparation was more monodisperse, which led to improvements in catalytic activity compared with the catalysts synthesized via the impregnation method (that achieved particle sizes of 2.2 nm in contrast) [77]. 

**Figure 4:**
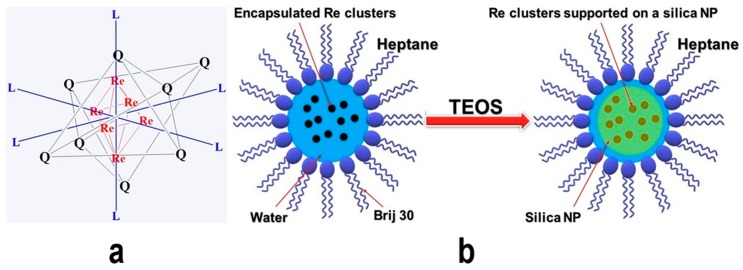
A schematic representation of (**a**) Re_6_Q_8_L_6_ cluster unit, and (**b**) the process of Re_6_Q_8_L_6_@SiO_2_ NPs encapsulation (as reported by Aubert et al. [81]), redrawn by the authors from the reference.

**Figure 5:**
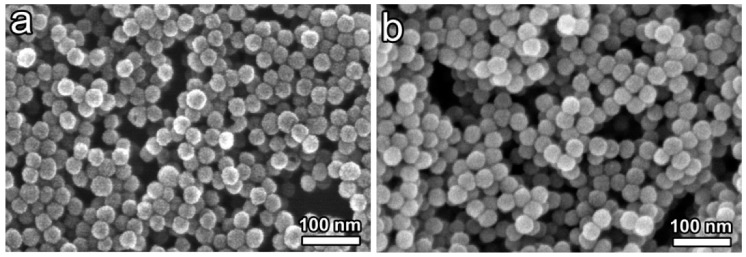
Field electron scanning electron microscopy (FE-SEM) of A_4_[Re_6_Q_8_L_6_]@SiO_2_ NPs, (**a**) K_4_[Re_6_S_8_(OH)_6_]@SiO_2_ (**b**) Cs_1.68_K_2.32_[Re_6_S_8_CN_4_(OH)_2_]@SiO_2_ NPs, Reprinted with permission from [81] (*Langmuir* **2010**, *26*, 18512–18518). Copyright (2010) American Chemical Society.

In terms of small cluster species, Aubert et al. in 2010 synthesized some stable [Re_6_ clusters@silica] NPs using a reverse microemulsion technique and evaluated their luminescence properties for biological purposes. The Re_6_ cluster compounds emit red and near-infrared (NIR) radiations, which are very useful for biotechnological applications. Firstly, they prepared some Re_6_ cluster units with the A_4_[Re_6_Q_8_L_6_] formula such as K_4_[Re_6_Se_8_(OH)_6_] and Cs_4_[Re_6_S_8_Br_6_] at high temperatures [78,79,80]. Then, they reported a preparation of Re_6_ cluster units encapsulated in the nanodroplets of a reverse microemulsion consisting of n-heptane/polyoxyethylene-4-lauryl ether (Brij 30) in an aqueous solution of Re_6_ cluster and ammonia. Afterward, the silica precursor was added (Figure 4) to the solution to synthesize the A_4_[Re_6_Q_8_L_6_]@SiO_2_ NPs (spherical 30-nm particles) which had been characterized by field electron scanning electron microscopy (FE-SEM) (Figure 5) and scanning transmission electron microscopy in annular dark field mode (ADF-STEM). Interestingly, the authors found that the luminescence of these NPs is not quenched by oxygen in aqueous solutions and the NPs kept their luminescence properties after encapsulation and embedding in silica. If toxicological studies confirm their overall safety, then they will be useful in biological applications [81].

In 2011, Kolb et al. introduced a high-throughput synthesis method of some metal and mixed metal sulfide NPs (some of which involved Re and Ir species). Initially, they made and set up a high-throughput device with a 9-fold reactor for a parallel preparation and purification of the sulfide NPs. Then, a reverse microemulsion of cyclohexane (as the oily phase), a mixture of non-ionic surfactants, NP5 and NP10 (nonylphenol polyethylene (5 and 10) glycol ether) or NP10 and Triton X-45 (4-(1,1,3,3-tetramethylbutyl) phenyl polyethylene glycol), and a water solution of some metal precursors (such as Re, Ir, and Rh) were applied for this purpose. They synthesized a number of amorphous (as determined via XRD studies) noble metal sulfide NPs in different phase compositions such as Re_34_S_66_, Rh_40_S_60_, Ir_21_S_79_, Ru_39_S_61_, and Au_38_S_61_ (as characterized by XRF) by the pressurization of the microemulsion solution with H_2_S gas as a high-throughput synthesis method, and claim that this method can be used for synthesizing other nanomaterials on a large scale [82]. Table 1 summarizes these recent advances in microemulsion-based synthesis of Re NPs.

### 2.2. Microemulsion-Based Methodologies for Generating Ir NPs

**Figure 6:**
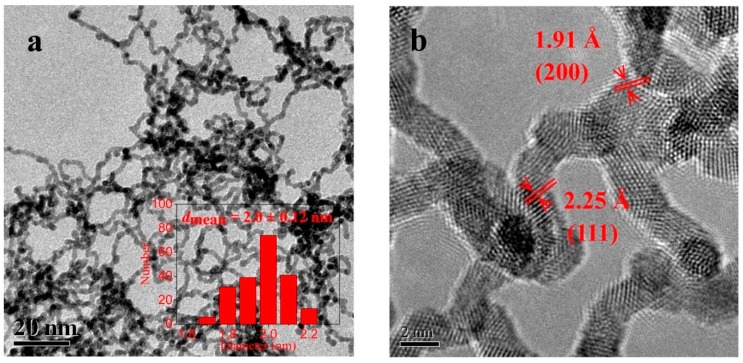
(**a**) TEM and (**b**) HRTEM images of the cetyl trimethyl ammonium bromide- (CTAB)-microemulsion synthesized Ir nanowires. Reprinted with permission from [83] (*Langmuir* **2015**, *31*, 90–95). Copyright (2014) American Chemical Society.

Lu et al. have recently prepared 1D iridium nanowires with the length of hundreds of nm in a number of different microemulsion media for the first time. Firstly, they prepared a transparent microemulsion solution using n-heptane/water/n-hexanol/surfactant and then, they added an Ir precursor (H_2_IrCl_6_) and the reductant (NaBH_4_) dropwise to this mixture to produce a black precipitate consisting of the Ir nanowires (average diameter 2 nm). The best quality Ir nanowires were achieved by using CTAB as the surfactant (1.8 nm Ir nanowires with no individual existence of discrete NPs). By decreasing the positive charge intensity of the surfactant polar heads to the non-ionic surfactants (e.g., from CTAB to Brij 30), the formation of nanowires decreased, and the generation of discrete nanoparticles increased. The Ir nanowires demonstrated common face-centred cubic (fcc) crystals with the (111) and (200) facets in a 3:1 ratio as characterized by X–ray diffraction (XRD) and HRTEM (Figure 6). Further characterization revealed that the concentration of reducing agent and surfactants have a considerable effect on orienting Ir NPs to form into nanowires (e.g., a higher concentration of CTAB was found to be desirable for this purpose). The synthesized Ir nanowires demonstrated a very high conversion rate and superb selectivity in catalyzing the hydrogenation of o-chloronitrobenzene to o-chloroaniline [83]. 

In 2017, Yang et al. reported a similar microemulsion-based synthesis of 1D Ir nanocrystals to that of Lu et al., and evaluated their electrocatalytic activity in the hydrogen oxidation reaction/hydrogen evolution reaction in an alkaline medium (0.1 M KOH). They used IrCl_3_/CTAB/n-C_6_H_14_/n-C_6_H_12_OH and NaBH_4_ (as reductant) to prepare the worm-like Ir nanocrystals which had lengths of tens or hundreds of nm (as characterized by TEM studies). XRD and HRTEM studies also revealed the fcc lattices of the prepared Ir nanocrystals. The surfactant concentration had a critical role in dictating the Ir nanocrystal morphology which given insufficient CTAB presence led to no 1D Ir NPs being observed. The synthesized 1D Ir nanocrystals showed higher electrocatalytic activity (because of their larger active surface area and faster mass/charge transfer, and lower hydrogen binding energy which was facilitated by the presence of long segment low-index crystalline planes) for the above-mentioned hydrogenation reactions in comparison with 0D Ir NPs and close activity to commercially available Pt/C NPs [84]. 

In 2010, Zhang et al. prepared an organoiridium(III) complex NPs by encapsulating the Ir complex in a CTAB microemulsion as a template. The iridium(III) bis (2-phenylbenzothiozolato-N, C^2’^) acetylacetonate, (BT)_2_Ir(acac), was used as the precursor of the Ir complex. The CTAB in water generated normal micelles, which encapsulated the organoiridium complex in the hydrophobic interior core. After encapsulating the Ir(III) complex sphere-like NPs in the micelles, a perylene perchlorate precursor was added to the solution and was reduced by the water which exists outside the Ir complex NPs. Consequently, they obtained core-shell nanoparticles of (BT)_2_Ir(acac)@perylene (as proved by EDX) with a 30 nm average diameter of the core particles (as characterized by TEM) to prepare NPs for a triplet-triplet annihilation-based upconversion. The core of the synthesized NPs would act as a synthesizer (excited with green light) and the shell would act as a triplet annihilator (blue emission). The emission light could be changed from orange to blue by adjusting the Ir complex to perylene molar ratio [85]. 

**Figure 7:**
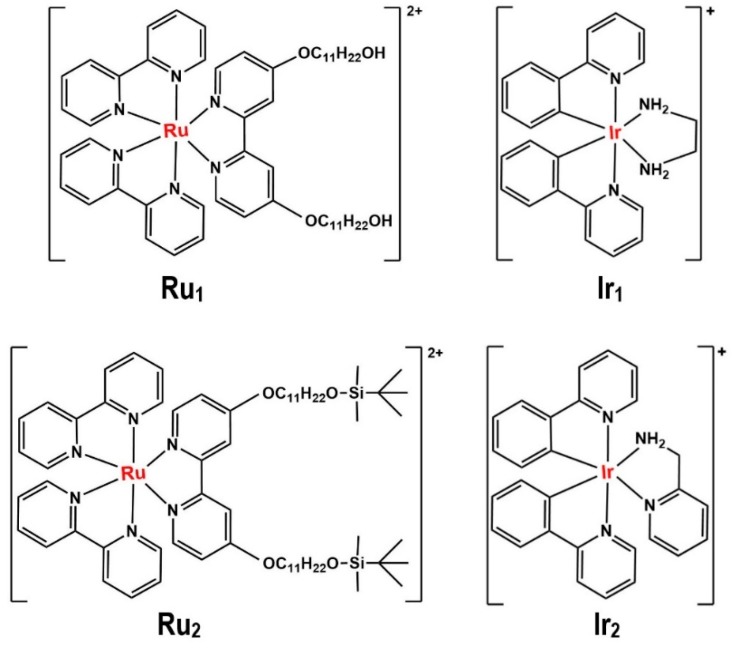
Chemical structures of Ru_1_, Ru_2_, Ir_1_, and Ir_2_ complexes used by Ricciardi et al. [86], redrawn by the authors from the reference.

Another microemulsion-assisted synthesis of Ir complex NPs has been done by Ricciardi et al. in 2014. They synthesized some Ir(III) and Ru(II) complexes@gold-silica NPs (“M@GS-NPs”) by a reverse microemulsion technique with potential biomedical applications (e.g., theranostic purposes). As shown in Figure 7, the selected Ru(II) and Ir(III) complexes were two bipyridine (bpy)-derived ligands (as Ru_1_ and Ru_2_) and two phenylpyridine (ppy) ligands (as Ir_1_ and Ir_2_). To synthesize the Ru_1_, Ru_2_, Ir_1_, and Ir_2_@GSNPs, they first prepared the Au NPs by a W/O microemulsion containing cyclohexane (oil phase)/Triton X-100 (surfactant)/n-hexanol (co-surfactant) and added an aqueous solution of HAuCl_4_.3H_2_O (gold precursor), NaBH_4_ (reductant), and sodium 2-mercaptoethanesulfonate to the microemulsion system. Then, they added an aqueous solution of an Ir(III) complex or a DMSO solution of Ru(II) complexes, followed by the tetraethoxysilane (TEOS), (3-aminopropyl)triethoxysilane (APTES), and aqueous ammonia addition. Also, N-(3-triethoxysilyl) propylsuccinic anhydride was used to protect and increase the electrostatic stability of the synthesized NPs in these systems. TEM and DLS measurements revealed the average size (TEM) to be 50 ± 2 nm and the hydrodynamic diameter (DLS) to be 60 ± 2 nm for the Ru_1_@GSNPs (as a representative sample of all the M@GSNPs made) [86].

In 2014, Felix-Navarro et al. reported the synthesis of a metallic and a bimetallic form of Ir, Pt, and Pt-Ir NPs supported on multiwall carbon nanotubes (MWCNTs) via a microemulsion medium and evaluated their electrocatalytic activity for hydrogen peroxide production by the reduction of oxygen molecules. To prepare Pt or Ir NPs, they used a mixture of two microemulsion solutions of (1) Brij30/hexane/isopropanol/MWCNTs; and (2) a metal precursor (IrCl_3_ or K_2_PtCl_6_) and added a 1:1 ratio of ascorbic acid and sodium citrate (as the reducing agents) to the mixture to reduce (with heating) the metal ions for preparing the bimetallic NPs. The preparation process for the bimetallic Pt-Ir@MWCNT NPs involved, initially, the Pt NPs formation via the aforementioned microemulsion system and reductants. Then, the Ir precursor with the second dose of the reductants was added to these Pt NPs to obtain the bimetallic NPs. TEM studies proved that the metallic and bimetallic NPs were anchored onto the MWCNTs. The electrocatalytic performance (as catalyst) in regard to H_2_O_2_ generation demonstrated that the Pt-Ir@MWCNT NPs catalyse the H_2_O_2_ generation process more efficiently than the individually prepared Pt or Ir@MWCNTs NPs [87]. Szumelda et al. have also used a reverse microemulsion system to synthesize some monometallic and bimetallic (Pd-based) noble metal NPs supported on carbon (with 2 wt% of total metal loading) including Ir/C, Ru/C, Pd-Ir/C, and Pd-Ru/C NPs (with a molar ratio of 10:1 for Pd:M bimetallic NPs). The microemulsion composition was cyclohexane/polyoxyethylene (7-8) octylphenyl ether (Triton X-114) and an aqueous solution of the metal precursor(s) (*W* value = 5.5). The reducing agent (NaBH_4_) was added to the microemulsion directly (the reductant to the metal ions molar ratio = 10) in order to prepare the metal particles which manifested as a black solution. Carbon black and THF were subsequently introduced into the solution to allow deposition of the metal particles onto the carbon (eventually yielding a colorless solution of settled black materials). Electron microscopy (Scanning Electron Microscopy (SEM), TEM, and HRTEM), X–ray Photoelectron Spectroscopy (XPS), and XRD were used for morphological and structural studies of these supported NP systems and confirmed that the NPs were supported on carbon. The studies demonstrated that the Ir/C and Pd-Ir/C NPs had the smallest average size (4.2 and 4 nm, respectively) with the common fcc structure observed for the Ir/C NPs [88]. Table 2 summarizes these recent advances in microemulsion-based synthesis of Ir NPs.

### 2.3. Microemulsion-Based Methodologies for The Generation of Rh NPs Synthesis

Sanchez-Dominguez et al. have prepared some metal and metal oxide NPs such as those of Rh by an uncommonly used microemulsion method. They claimed that given the high number of oil phases employed for preparing W/O microemulsion systems can give rise to some environmental and industrial usage problems, applying a normal microemulsion system (which used water as the dispersion phase) and also selecting some biodegradable surfactants for synthesizing metal and metal oxide NPs, can be more practical and eco-friendly. They used water/polyoxyethylene (10) oleyl ether (Brij 96V as a non-ionic surfactant)/butyl-s-lactate as the normal microemulsion system and bis(1,5-cyclooctadiene) dirhodium(I) dichloride (Rh-COCl) as the organometallic precursor of Rh dissolved in an oily phase, and added to this an aqueous solution of NaBH_4_ (with a 2:1 or higher molar ratio of the reductant to the metal) to form the Rh NPs. TEM samples were prepared (as reported) via sonication of one drop of the Rh NPs microemulsion solution in isopropanol and deposition onto the carbon copper grid. Small particles (3.9 nm) with narrow particle size distributions were observed for the synthesized Rh NPs as determined by TEM measurements [89]. 

Yoon et al. have used a reverse microemulsion of hexane/AOT/a solution of the metal precursor(s) to synthesize some metal (Pd, Pt, Au, Rh) and Pd/Rh bimetallic NPs supported on carboxylic acid functionalized MWCNTs (*W* = 12). They used H_2_ gas as the reducing agent to obtain the CNT-supported metal NPs and tested these catalysts for their ability to hydrogenate benzene and its derivatives (in the trials, Pd-Rh/CNT demonstrated the highest catalytic activity compared to the other prepared catalysts). The average particle size distribution of the Rh/CNT and Pd-Rh/CNT NPs was determined by TEM to be 5.6 ± 1.3 nm and 4.6 ± 1 nm, respectively [90]. A study by Liao et al. in 2011 reported the synthesis of Pt, Rh, and Pt-Rh NPs supported on phenylacetic acid-modified single-wall carbon nanotubes (SWCNT). The same method and reagents (which Yoon et al. used) were applied for the Pt, Rh, and Pt-Rh/CNT NPs preparation. TEM studies revealed that the average particle size of the NPs was 4.5 ± 1.4 nm for Rh and 2.3 ± 0.6 nm for Pt-Rh NPs. The synthesized NPs supported on the SWCNT displayed much higher catalytic activity than the commercially available Pt/C and Rh/C catalysts for hydrogenation of benzene at room temperature (the bimetallic NPs showed the highest activity) [91].

**Figure 8:**
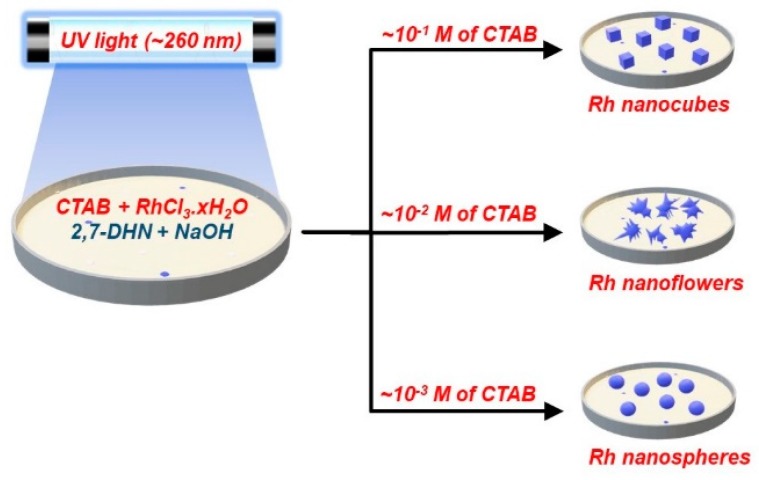
The Kundo et al. [92] micellar-media synthesis method for generating different shapes of Rh NPs depending on the CTAB surfactant concentrations, redrawn by the authors from the reference.

**Figure 9:**
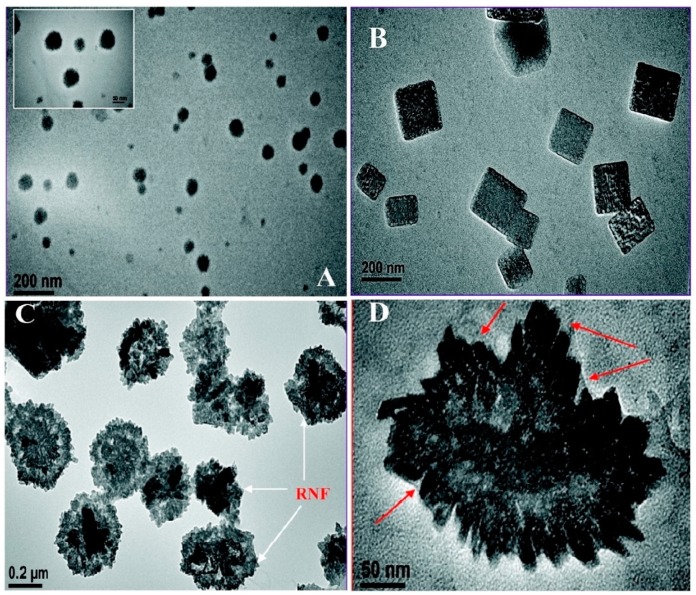
TEM images of (**A**) Rh nano spheres; (**B**) Rh nanocubes; (**C**) High-magnification image of Rh nanoflowers; (**D**) Image of a single Rh nanoflower, all synthesized by Kundu et al. [83], Reprinted with permission from [92] (*J. Phys. Chem. C* **2009**, *113*, 18570–18577). Copyright (2009) American Chemical Society.

Kundo et al. reported an interesting, micellar-based study in which different shapes of Rh NPs were realized by merely altering the surfactant to metal ion ratio in a micellar-based solution subject to doses of UV-irradiation to effect the metal ion reduction. They used a CTAB, Rh(III) solution, and an alkaline 2,7-dihydroxynaphthalene (2,7-DHN) mixture to produce Rh seeds and then irradiated it under UV light (at 260 nm which is close to the UV absorption band of 2,7-DHN) for 6h to grow the particles and to generate nanostructures of Rh (as nanocubes, nano spheres, and nanoflowers). NaOH was used to increase the pH of the solution and so boost the strength of the reducing activity of 2,7-DHN. During the UV-photoirradiation, hydroxyl radicals generated from the alkaline reductant (2,7-DHN has acidic OH groups) reduced the Rh(III) ions to metallic Rh (as confirmed by XPS). High concentrations of CTAB (high molar ratio of CTAB to metal precursor) formed cubic Rh NPs while a low amount of CTAB led to spherical Rh NPs. When the molar ratio of CTAB to the metal ion was at intermediate values, the Rh NPs adopted a florette-type morphology (Figure 8). TEM, XPS, and XRD studies revealed the morphology, size, and chemical identity of the particles. TEM images (Figure 9) showed Rh nanocubes (of 160 ± 10 nm average side length), nano spheres (of 60 ± 5 nm diameter), and nanoflowers (of 250 ± 50 nm diameter and ~10 nm width of the flower-like Rh particles). Evaluation of the catalytic performance of the different-shaped synthesized Rh NPs with respect to the NaBH_4_-reduction of 4-nitroaniline to paraphenylene diamine showed very good catalytic activity on the cubic Rh NPs [92].

Montes et al. synthesized some precious metal NPs (of Au, Pt, Pd, and Rh) by a reverse microemulsion technique which involved their deposition onto ZnO functioning as the support, and used this system for catalyzing the formation of 1,2-propanediol (1,2-PDO) and 1,3-propanediol (1,3-PDO) via glycerol dehydrogenation. They synthesized Rh NPs by mixing a non-ionic surfactant (Synperonic 13/6.5) with the oil phase (trimethylpentane) and adding a water solution of the metal precursor to obtain a transparent mixture at room temperature (they tried to use a small amount of surfactant and an oily phase to reduce the environmental impacts of such systems). Subsequently, they deoxygenated the system by bubbling in nitrogen gas and adding a reducing agent (NaBH_4_ or N_2_H_4_) in a 1:5 molar ratio of metal precursor to reductant under an inert atmosphere to prepare the Rh NPs (the color of the reaction solution changed from red to black after adding the reductant). To induce the deposition of the prepared NPs onto ZnO, they used a destabilizing agent (acetone) and added the support to the solution mixture. TEM studies demonstrated that the NaBH_4_-reduced NPs of all metals were smaller than the NPs obtained by hydrazine reduction (however, N_2_H_4_ was not able to reduce the Rh salts to Rh metal by this method). Rh-supported NPs had the highest reactivity amongst all the metal-supported NPs synthesized and tested in the paper reported by Montes et al. They also compared the glycerol dehydrogenation catalytic activity of NPs synthesized through a microemulsion technique with NPs prepared via a deposition-precipitation (DP) method. The microemulsion NPs showed a comparatively higher selectivity and lower activity in comparison with DP-systems (even though smaller particles were obtained through the microemulsion technique). They assumed that the remaining surfactants (after heating and removing surfactants) might have caused the activity loss [93].

Two Russian teams (Sergeev et al. in 2014 and Revina et al. in 2015) reported the preparation of mono-metallic and bimetallic Ru, Rh and Pd NPs using a reverse microemulsion system via the methods previously described for synthesizing Re NPs by Revina et al. [64,65] Sergeev et al. synthesized Pd, Rh, Pd_core_/Rh_shell_, Rh_core_/Pd_shell_, and substitution alloy of Pd-Rh NPs supported on γ-alumina. They used a microemulsion system of isooctane/AOT/metal salt(s) and reduced the metal ions by two methods: (1) a chemical reduction (using quercetin as a reductant); and (2) a radiation chemical method. For synthesizing the bimetallic NPs, a solution of the core metal precursor was firstly added to the system and reduced with the mentioned methods then, the second metal precursor as the shell added (with a 1:1 molar ratio of metals) to the mixture. To generate the substitution alloy of the Pd-Rh NPs, a 1:1 molar ratio of both metal aqueous solutions were mixed simultaneously. A range of W values for the synthesis of mono-metallic particles was evaluated as well and proved the expected result of larger NPs being obtained at higher *W* values (for the bimetallic NPs only *W* = 5 was used). AFM studies demonstrated a small average particle size with a narrow particle size distribution of (0.7–6.5 ± 1.5 nm) for the prepared NPs. They applied the synthesized NPs for two catalytic reactions, homomolecular isotope exchange of hydrogen gas and ortho-para protium conversion. The Pd_core_/Rh_shell_ NPs exhibited a four-fold higher catalytic activity (a synergistic effect from having both metals) compared to the mono-metallic NPs in the molecular hydrogen reaction [94]. Subsequently, Revina et al. applied the same methods (as Sergeev et al.) for preparing Pd, Ru, and Rh NPs and supported them on γ-alumina for some catalytic applications. They investigated the optical properties of the particles and observed that the prepared Rh NPs quench the luminescence properties of the micellar solution with the opposite occurring for the Pd and Ru NPs. As calculated from the AFM images, a mean particle size of 2–4 nm was obtained for Rh NPs at the smallest *W* value used (*W* = 1). They also observed that smaller particles were prepared at a lower dose of radiation when using the gamma irradiation method. The synthesized Ru and Rh NPs@γ-Al_2_O_3_ exhibited a high catalytic activity in the aforementioned isotope exchange reaction of hydrogen over a wide range of temperatures [95]. 

Guo et al. synthesized Rh NPs (that were 10 nm in particle size as characterized by TEM) by mixing equal amounts of two AOT-2,2,4-trimethylpentane microemulsion systems containing an aqueous solution of RhCl_3_ and NaBH_4_. They deposited the resultant NPs onto a Pt electrode electrophoretically by immersing the Pt electrode into the Rh NPs microemulsion solution and applying a 100 V/mm electric field at the electrode (SEM images showed a two-fold increase in particle size of the Rh NPs after deposition on the Pt electrode). After other modifications, the electrode was found to be highly sensitive and selective with respect to glucose biosensing functionality in real blood samples [96].

In another report, Suryawanshi et al. prepared Rh NPs@γ-Al_2_O_3_ via two different techniques, a non-microemulsion microwave (MWv)-assisted method and a microemulsion-based technique. The Rh NPs obtained by the microemulsion-based method were smaller (which had consequently a higher catalytic activity) compared to the MWv-generated Rh NPs. The microemulsion synthesis process involved the mixing of two cyclohexane/AOT microemulsion systems of aqueous RhCl_3_ solution and aqueous NaBH_4_ (reductant) solution at the same *W* values (equal to three). The MWv-assisted synthesis was carried out using a microwave power of 300 W, at 200 °C when reacting RhCl_3_ and PVP (as the protecting agent), dissolved in ethylene glycol (as solvent and reductant). Preparation of the Rh NPs was monitored by UV-Vis spectroscopy and detected by quenching the absorbance peak of Rh(III) which proved that the reduction of Rh(III) to Rh(0) had occurred. DLS studies showed a 4–14 nm hydrodynamic radius (unsupported particles) for both microemulsion-generated and MWv-synthesized Rh NPs but further TEM analysis revealed that a size range of 2–10 nm (average size ~4 nm) was observed for the microemulsion-based Rh@γ-Al_2_O_3_ NPs and 4–14 nm (average particle size ~9 nm) for the MWv-generated Rh@γ-Al_2_O_3_ NPs. They applied the supported nanocatalyst systems to catalyze the hydrogenation of 4’,4”(5”)-di-tert-butyldibenzo-18-crown-6 ether. The microemulsion-synthesized nanocatalysts exhibited higher activity in comparison to the MWv-generated nanocatalysts [97].

**Figure 10:**
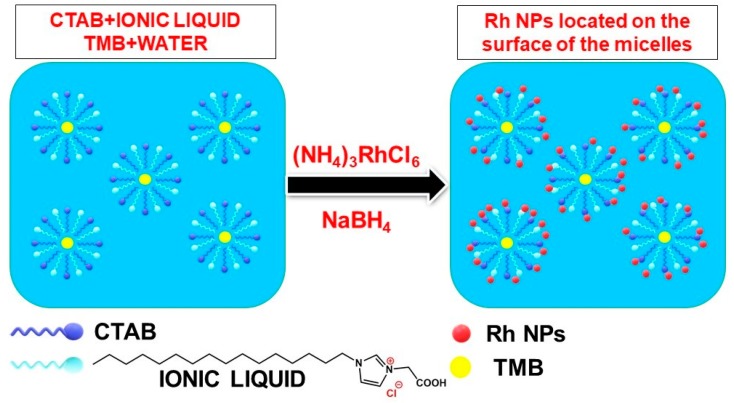
Synthesizing Rh NPs by a CTAB-ionic liquid micellar template (Yao et al. [98]), redrawn by the authors from the reference.

Yao et al. synthesized very small Rh NPs using a normal micelle template with a water-soluble ionic liquid which improved the micellization and the size control of the particles (Figure 10). They used a system consisting of a CTAB/ionic liquid/water solution of (NH_4_)_3_RhCl_6_ in a trimethylbenzene (TMB) oil and NaBH_4_ as reductant. They first mixed the CTAB, ionic liquid and TMB to form a normal micelle structure. Then, they added the Rh metal salt precursor and the reductant to produce small Rh NPs in the 1–2 nm size (as characterized by TEM). The prepared Rh NPs exhibited high catalytic activity in a green chemistry reaction involving the synthesis of aldehydes and ketones via the oxidation of alcohols in pure water [98]. 

**Figure 11:**
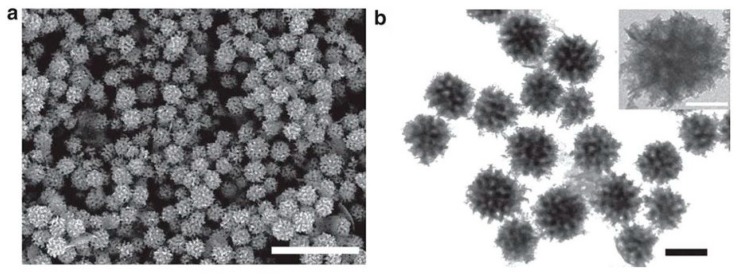
(**a**) SEM (scale bar 500 nm) and (**b**) TEM (scale bar 100 nm) images of mesoporous Rh NPs, reprinted from the [99] under a Creative Commons Attribution 4.0 International License.

An interesting article has also been published by Jiang et al. on the micellar-mediated synthesis of mesoporous Rh NPs that have a very high thermal stability (up to 400 °C). They claimed to have prepared mesoporous Rh NPs by a chemical reduction method for the first time. A mixed solvent consisting of water and N,N-dimethylformamide (DMF) along with Na_3_RhCl_6_/poly(ethylene oxide)-b-poly(methyl methacrylate) (PEO-b-PMMA) [b = block copolymer], and ascorbic acid as the reductant were used to generate the mesoporous Rh NPs. The synthesis involved firstly the dissolution of the PEO-b-PMMA in DMF with the subsequent addition of water to form the micelle templates and then mixing of the metal precursor salt with the reducing agent to generate the initial Rh nuclei. These were then grown to form the mesoporous structure of the Rh NPs. The synthesized particles were analyzed by several characterization methods such as SEM, TEM (Figure 11), and XRD to confirm the morphology and structure of the mesoporous Rh NPs (these were of ca. 100 nm average particle diameter as estimated by SEM analysis). These nanoparticles exhibited a 2.6 x higher electrocatalytic activity (as catalyst) with respect to the methanol oxidation reaction relative to commercial Rh catalysts available. They also assisted in removing nitric oxide (NO) in automobile exhausts under lean-burn conditions [99].

Very recent research by Zahedifar et al. used a microemulsion system of cyclohexane/1-pentanol/cetylpyridinium bromide (CPB) together with an aqueous solution of Na_3_RhCl_6_/ascorbic acid and heated these to 100 °C to synthesize mesoporous Rh NPs with a fibrous morphology. CPB acted as a cationic surfactant to form the reverse micelles in the microemulsion system. SEM studies demonstrated the high thermal (up to 500 °C) and mechanical (up to 120 MPa) stability of the synthesized Rh NPs which had a dendritic fiber structure of about 12 nm thickness. Also, TEM images revealed the significant role of ascorbic acid concentration on the morphology and the size of the Rh NPs. At low ascorbic acid concentration (60 mM), spherical NPs formed while at high concentrations of ascorbic acid (100 mM) highly fibrous Rh NPs were generated. These particles exhibited a high catalytic activity in the reaction involving the reduction of carbon dioxide to form formate with a five-cycle reusability observed (with no activity loss) [100]. 

Cao et al. synthesized Pt-Rh@BHA (barium hexa-aluminate) NP systems with precise compositional control of high thermal stability via a reverse microemulsion system. They used a microemulsion system of iso-octane/pentanol/poly(propylene glycol)-block-poly(ethylene glycol)-block-poly(propylene glycol) (PPG-b-PEG-b-PPG) as the surfactant together with an aqueous solution of H_2_PtCl_6_ and RhCl_3_ and mixed it with a solution of aluminum isopropoxide and barium isopropoxide dissolved in iso-propanol to prepare the bimetallic Pt-Rh@BHA nanocatalysts. This method had previously been applied by these authors for the preparation of Pt@BHA nanocomposites [101,102]. As characterized by TEM, the bimetallic NPs (with a 1:1 Pt/Rh ratio) had a 4.1 ± 0.5 nm average size. They observed no size change for the synthesized bimetallic nanocatalysts after calcination to 850 °C. This high thermal stability depended on the Pt/Rh ratio. The Pt content of the nanocomposites was responsible for the catalytic activity while the Rh content was responsible for the thermal stability. The synthesized nanocatalysts exhibited high catalytic activity in the methane combustion reaction [103]. 

Rh doped ceria NPs have a variety of catalytic applications e.g., in fuel cell reactions and in three-way catalysts (TWCs) [104,105,106,107,108,109] (TWCs are used to decrease the noxious exhaust molecules from internal combustion engines by converting hydrocarbons, CO, and nitrogen oxides to the more innocuous molecules of CO_2_, and N_2_ [22,33]). Kurnatowska et al. synthesized Ce_1−x_Rh_x_O_2−y_ mixed oxide nanocrystals (average size of 4–5 nm) via a microemulsion system of cyclohexane/triton X-100/1-pentanol and an aqueous solution of Ce and Rh nitrates. After some treatments and purifications, the achieved precipitated oxide powder was dried and heated at 500 °C in oxygen. Using this method, they prepared a range of mixed oxide nanocrystals with a wide range of Rh doping levels (x = 0.03–0.21, as confirmed by EDS) which had been noted previously to be very low in equivalent systems made using other non-microemulsion-based synthesis methods (x = 0.005–0.02). Also, they investigated the structural and thermal stability of the different compositions of the nanocrystals (x = 0–0.16) by heating the samples in hydrogen and oxygen (reducing and oxidizing) atmospheres respectively, up to 1000 °C. It revealed a strong thermal and structural stability enhancement (depending on the doping level, x) of the synthesized mixed oxide nanocrystals compared to undoped pure CeO_2_ NPs. For example, heating Ce_0.89_Rh_0.11_O_2−y_ in oxygen at 500 °C and 800 °C demonstrated no phase separation (no existence of discrete Rh oxide phases) and a small and limited increase in size (from 4.2 nm to 8.8 nm) of the Ce_0.89_Rh_0.11_O_2−y_ samples while (purely) CeO_2_ NPs displayed a much higher growth in size (from 8.6 nm to 56 nm) when subjected to the same heating conditions [110]. Table 3 summarizes these recent advances in microemulsion-based synthesis of Rh NPs.

## 3. Conclusions and Recommendations for Future Investigation

It has been shown that both the W/O and O/W microemulsion-based preparation methodologies can be successfully applied for synthesizing a number of metal nanostructures (in multiple forms being metallic, oxide, multi-metal, and discrete molecular complex forms), particularly for those NP systems that are highly unstable and have a strong tendency to aggregate if prepared via conventional non-microemulsion-based methods. The merits of using this technique relative to other colloid preparation techniques are: a) the ability to more closely control the size of particles; b) to produce more monodisperse particle size distributions; c) to be able to change the morphology and composition of the particles; and d) the ability to colloidally stabilize the particles. The only considerable disadvantage of this route may be the high amount of surfactants (and co-surfactants) used which can raise some concerns about separation of them from the NPs, the environmental effects of the surfactants/oils and the financial cost of utilizing these systems, especially when considering their manufacture on an industrial scale. So, further investigation into the possibility of synthesizing larger amounts of nanomaterials by using a lower concentration of surfactants should be considered as a strategy in future microemulsion-based synthesis research. It might need some optimization studies, since the lower the surfactant concentration, the higher the *W* values, and generally bigger particles will result, which is not a desirable outcome. The other suggestion that others in the field could consider is further research into the employment of safer, cheaper, and eco-friendlier microemulsion systems which can be industrialized or applied for biomedically targeted uses. 

In addition, exploiting the capacity of the microemulsion technique for synthesizing and stabilizing NPs, and widening the catalytic applications of the noble metals and increasing research efforts into the field of microemulsion-based synthesis of noble metal NP systems (with more focus on the less studied noble metals discussed in this review) will be crucial for developing this important nanotechnology. Surprisingly, it has been seen that the microemulsion synthesis of Os nanomaterials has attracted the least attention (among the other evaluated metals) so far and, therefore, further efforts are needed to study ways of generating NPs of this metal using the microemulsion approach. Also, as seen in recent microemulsion investigations, mixed metal NP/nanomaterial systems can usually generate materials with multiple beneficial properties (e.g., doping Rh into other metal oxide systems like ceria generates NPs of higher thermal stability). So, further microemulsion synthesis investigations on mixed metal or mixed metal oxide systems which includes at least one of the Re, Rh, Ir, or Os metals, and evaluating their properties can further develop the potential nanotechnological applications of these materials to a more sophisticated extent.

## Figures and Tables

**Table 1:** A summary of recent advances in microemulsion-based synthesis of Re nanoparticles (NPs). DDAB—dimethyl dioctyl ammonium bromide; DDAI—dimethyl dioctyl ammonium iodide; AOT—dioctylsulfosuccinate sodium.

Metal NP Composition	Microemulsion Type	Surfactant	Particle Size	Highlight(s) of Synthetic Method	Ref
Re NPs	Reverse microemulsion (a/o)	DDAB or DDAI	2.2 nm	Useful for synthesizing oxidation-sensitive NPs. Needs very low temperature	[72]
Re NPs	Reverse microemulsion	AOT	1–18 nm (depends on the synthesis conditions)	Control of the particle size with varying γ-irradiation doses.	[73,74]
Re/Re oxide NPs	Reverse microemulsion	AOT	0.7–1.4 nm	Obtained very small sized particles with a narrow size distribution but metal NPs oxidation state was not studied. Reverse trend observed for W factor effect.	[75]
Pt-Re bimetallic NPs	Reverse microemulsion	Triton X-100	1.8–2.05 nm	Small bimetallic NPs obtained, Used a very low *W* value (=0.3)	[77]
Re6 cluster@SiO2 NPs	Reverse microemulsion	Brij 30	30 nm	NPs kept their luminescence properties in aqueous solutions (potential for biological applications) (need to form multinuclear Re cluster at high temperatures first)	[81]
Re sulfide NPs	Reverse microemulsion	NP5/NP10 or NP10/Triton X-45	-	Introduced a novel high-throughput microemulsion synthesis device	[82]

**Table 2:** Recent development in microemulsion-based synthesis of Ir NPs. CTAB—cetyl trimethyl ammonium bromide.

Metal NP Composition	Microemulsion Type	Surfactant	Particle Size	Highlight(s) of Synthetic Method	Ref
Ir nanowires	Reverse microemulsion	Several surfactants such as CTAB, Triton X-100, Brij 30, etc.	1.8 nm diameter (for the CTAB-micelles)	Presents a facile method for synthesizing 1D Ir nanomaterials and evaluating the effects of different surfactants on the morphology and size of prepared Ir NPs	[83,84]
Organoiridium (III) complex NPs	Normal microemulsion	CTAB	30 nm	Uses an organometallic complex for a normal microemulsion NP synthesis. Reduction of perylene (which acts as a triplet annihilator) occurs outside the CTAB-micelles with the formation of core-shell Ir complex@perylene NPs	[85]
Ir(III)complex@GS NPs	Reverse microemulsion	Triton X-100	50 nm for Ru_1_@GSNPs (as a sample of all the M@GSNPs)	Synthesizes doped transition metal complexes into GS NPs with potential biomedical applications	[86]
Ir and Pt-Ir bimetallic NPs anchored onto MWCNTs	Reverse microemulsion	Brij 30	-	Prepares a stable and reusable cathode of bimetallic Pt-Ir NPs@MWCNTs	[87]
Ir and Pd-Ir bimetallic NPs supported on carbon	Reverse microemulsion	Triton X-114	4 nm for Ir/C and 4.2 nm for Pd-Ir/C NPs	The size of mono- and bimetallic NPs has been studied. The synthesized Pd-Ir-0.1/C NPs were much smaller than the Pd/C NPs. Among all the mono- and bimetallic/C synthesized NPs prepared, the Ir mono- and bimetallic/C NPs turned out to be the smallest.	[88]

**Table 3:** Recent development in microemulsion-based synthesis of Rh NPs. CPB—cetylpyridinium bromide.

Metal NP Composition	Microemulsion Type	Surfactant	Particle Size	Highlight(s) of Synthetic Method	Ref
Rh NPs	Normal microemulsion	Brij 96V	3.9 nm	Synthesis of metal and metal oxide NPs including Rh by a normal microemulsion (less oil phase)	[89]
Rh mono- and bimetallic NPs supported on functionalized multi- and single wall CNTs	Reverse microemulsion	AOT	5.6 nm and 4.6 nm for Rh and Pd-Rh, and 4.5 nm and 2.3 nm for Rh and Pt-Rh, respectively.	The bimetallic supported NPs showed a significant increase in their catalytic activity compared to monometallic supported NPs	[90,91]
Rh NPs supported on ZnO	Reverse microemulsion	Synperonic 13/6.5	2.1 nm	The selectivity of the microemulsion-synthesis Rh@ZnO NPs increased with respect to glycerol dehydrogenation but the activity compared to DP-solids decreased which could be caused by the presence of some remaining surfactant molecules around the particles after heating	[93]
Rh mono- and bimetallic NPs	Reverse microemulsion	AOT	0.7–6.5 nm, and 2–4 nm	Smaller particles prepared by lower γ-irradiation doses	[94,95]
Rh NPs	Reverse microemulsion	AOT	10 nm	Prepared a very good Rh NPs-modified Pt electrode which performed as an effective glucose biosensor in real blood samples	[96]
Rh NPs	Reverse microemulsion	AOT	4 nm	Smaller particles with higher activity obtained by the microemulsion technique compared to MWv-method	[97]
Rh NPs	Normal microemulsion	CTAB	1–2 nm	Using an ionic liquid to improve the micellization and the size control of the particles	[98]
Rh NPs	Polymer-micellar template	PEO-b-PMMA	100 nm	Synthesizing mesoporous Rh NPs for the first time by applying a chemical reduction method	[99]
Rh NPs	Reverse microemulsion	CPB	12 nm	Synthesizing a fibrous-structure of Rh NPs with high thermal and mechanical stability and high surface area	[100]
Pt-Rh@BHA NPs	Reverse microemulsion	(PPG-b-PEG-b-PPG) polymer	4.1 nm	Prepared bimetallic Pt-Rh NPs with exceptionally high thermal stability and precise control of composition	[103]
Ce_1−x_Rh_x_O_2−y_ mixed oxide NPs	Reverse microemulsion	Triton X-100	4–5 nm	Prepared a range of Ce-Rh mixed oxide nanocrystals with a wide and higher range of Rh doping levels with their structural stability studied under oxidizing and reducing atmospheres	[110]
